# The distribution of the thermally tolerant symbiont lineage (*Symbiodinium* clade D) in corals from Hawaii: correlations with host and the history of ocean thermal stress

**DOI:** 10.1002/ece3.556

**Published:** 2013-04-09

**Authors:** Michael Stat, Xavier Pochon, Erik C Franklin, John F Bruno, Kenneth S Casey, Elizabeth R Selig, Ruth D Gates

**Affiliations:** 1 The UWA Oceans Institute and Centre for Microscopy, Characterisation and Analysis, The University of Western Australia35 Stirling Hwy, Crawley, Western Australia, Australia, 6009; 2 Australian Institute of Marine Science35 Stirling Hwy, Crawley, Western Australia, Australia, 6009; 3 CSIRO Marine and Atmospheric ResearchPrivate Mail Bag 5, Wembley, Western Australia, Australia, 6913; 4 The Cawthron Institute98 Halifax Street East, Private Bag 2, Nelson, New Zealand, 7042; 5 Hawaii Institute of Marine Biology, School of Ocean and Earth Science and Technology, University of HawaiiKaneohe, Hawaii, 96744; 6 Department of Biology, The University of North Carolina at Chapel HillChapel Hill, North Carolina, 27599; 7 National Oceanographic Data Center, National Oceanic and Atmospheric AdministrationSilver Spring, Maryland, 20910; 8 The Betty and Gordon Moore Center for Ecosystem Science and Economics, Conservation InternationalArlington, Virginia, 22202

**Keywords:** Climate change, coral, ITS2, *Symbiodinium*, symbiosis

## Abstract

Spatially intimate symbioses, such as those between scleractinian corals and unicellular algae belonging to the genus *Symbiodinium*, can potentially adapt to changes in the environment by altering the taxonomic composition of their endosymbiont communities. We quantified the spatial relationship between the cumulative frequency of thermal stress anomalies (TSAs) and the taxonomic composition of *Symbiodinium* in the corals *Montipora capitata*, *Porites lobata*, and *Porites compressa* across the Hawaiian archipelago. Specifically, we investigated whether thermally tolerant clade D *Symbiodinium* was in greater abundance in corals from sites with high frequencies of TSAs. We recovered 2305 *Symbiodinium* ITS2 sequences from 242 coral colonies in lagoonal reef habitats at Pearl and Hermes Atoll, French Frigate Shoals, and Kaneohe Bay, Oahu in 2007. Sequences were grouped into 26 operational taxonomic units (OTUs) with 12 OTUs associated with *Montipora* and 21 with *Porites*. Both coral genera associated with *Symbiodinium* in clade C, and these co-occurred with clade D in *M. capitata* and clade G in *P. lobata*. The latter represents the first report of clade G *Symbiodinium* in *P. lobata*. In *M. capitata* (but not *Porites* spp.), there was a significant correlation between the presence of *Symbiodinium* in clade D and a thermal history characterized by high cumulative frequency of TSAs. The endogenous community composition of *Symbiodinium* and an association with clade D symbionts after long-term thermal disturbance appear strongly dependent on the taxa of the coral host.

## Introduction

Recent global warming has contributed to changes in species ranges and community composition (Walther et al. 2002; Perry et al. 2005; Hoegh-Guldberg and Bruno 2010). For corals, the warming of the oceans temperature has resulted in an increase in the frequency and magnitude of bleaching events (Hoegh-Guldberg 1999). Bleaching is the paling of the external coloration of corals that reflects the breakdown of their obligate endosymbiosis with dinoflagellates in the genus *Symbiodinium* (Hoegh-Guldberg and Smith 1989). Bleaching often precedes the death of corals, and widespread bleaching events have driven mass coral mortality in some regions of the world (Hoegh-Guldberg 1999). The rapidly changing ocean environment has potentially dire consequences in the near future for reef ecosystems and the IUCN recently estimated that one third of reef corals are under an elevated threat of extinction (Carpenter et al. 2008). A better understanding of how corals could adapt and survive through changing ocean conditions is critical to developing predictions of species composition in future reef ecosystems.

The genus *Symbiodinium* is genetically diverse comprising nine evolutionary lineages referred to as clades A–I (Pochon and Gates 2010). The taxonomic composition of endosymbiotic *Symbiodinium* in corals is broadly recognized as an important factor that contributes to the environmental threshold of the host coral (Baker 2003; Berkelmans and van Oppen 2006; Stat et al. 2006). For example, corals such as *Acropora* and *Pocillopora* spp. that harbor clade D *Symbiodinium* show a higher thermal tolerance and resistance to bleaching than conspecifics with symbiotic communities dominated by clade C (Rowan 2004; Berkelmans and van Oppen 2006; but see Abrego et al. 2008). In addition, there have also been reports of symbiont community shifts in corals to clade D on reefs that have recently experienced bleaching and high ocean temperatures (Baker et al. 2004; Rowan 2004; Berkelmans and van Oppen 2006; Jones et al. 2008). These observations point toward the potential importance of *Symbiodinium* clade D in corals' adaptive response to changes in the environment. However, depressed growth rates in juvenile corals associated with clade D *Symbiodinium*, as compared with conspecifics in symbiosis with clade C (Little et al. 2004), have raised questions about the long-term benefits and/or ecological implications of hosting different *Symbiodinium* strains (Stat et al. 2008a; Cantin et al. 2009; Mieog et al. 2009; Jones and Berkelmans 2010; Ortiz et al. 2013).

While some studies have shown that the abundance of *Symbiodinium* clade D in corals increases during thermal stress and during recovery following bleaching (Jones et al. 2008; LaJeunesse et al. 2009), others have shown that the symbiont community in corals do not change under such conditions (Thornhill et al. 2006; LaJeunesse et al. 2007; Costa et al. 2008; Stat et al. 2009a). These inconsistencies point to the importance of the magnitude and duration of the stress and host-specific responses, as factors that shape the *Symbiodinium* communities in corals during and following bleaching (Goulet 2006; Stat and Gates 2011). It has also been shown that while the *Symbiodinium* in corals can become dominated by clade D under stress, reversion back to the original population in the absence of stress occurs in subsequent years, a feature indicating that chronic temperature stress is required to maintain symbioses dominated by clade D (Thornhill et al. 2005). More recently, the effects of thermal stress on *Symbiodinium* communities in corals over longer periods of time using remote-sensing satellite data (i.e., tens of years) as opposed to shorter periods such as a bleaching event (i.e., 1–2 years) is an alternative approach to investigating how ocean temperature influences the community composition of *Symbiodinium*. Oliver and Palumbi (2009) used remote-sensing information on ocean sea surface temperature for 1998–2006 from NOAA's Pathfinder v5 satellite data and investigated whether the number of degree heating weeks (DHWs) correlated with a greater abundance of *Symbiodinium* clade D in Acroporid corals from American Samoa, Fiji, Palmyra Atoll, and the Philippines. Interestingly, they showed that while Fiji yielded the greatest number of DHWs from the regions investigated, clade D was absent, and was only found in American Samoa, an area that had experienced threefold less DHWs. In American Samoa though, clade D abundance was correlated with higher ocean temperatures. The authors' interpreted spatial differences in the correlation between clade D and the history of ocean thermal stress to other factors; notably, local environmental conditions linked to a region. Cooper et al. (2011) identified water clarity and sediment type as one local condition influencing the distribution of clade D in *Acropora* from the Great Barrier Reef, showing that sea surface temperature anomalies did not explain the abundance and distribution of *Symbiodinium* clade D alone.

In a global assessment by Selig et al. (2010) on the frequency of thermal stress anomalies (TSAs) using NOAA's Pathfinder v5 dataset, the Hawaiian archipelago was shown to have some of the lowest frequencies and shortest durations of thermal stress events in the Pacific, although it experienced relatively high magnitudes, between 1985 and 2005. Coral reef ecosystems in the Hawaiian archipelago are dominated by five coral species, and of these corals, *Porites lobata*, *Porites compressa*, and *Montipora capitata* are among the most widespread and abundant (Fenner 2005; pers. obs.). *Montipora* in the Pacific and in some areas of Hawaii associates with *Symbiodinium* in clades C and D, however, the latter is extremely rare in *Porites* in the Pacific and has only ever been reported in two colonies from Palau (Fabricius et al. 2004; LaJeunesse et al. 2004a; Stat et al. 2011; Franklin et al. 2012). The aim of this study was to determine whether a higher frequency of cumulative TSAs is correlated with a higher occurrence of *Symbiodinium* clade D in *Porites* and *Montipora* across the Hawaiian archipelago.

## Materials and Methods

### Sample collection

**Figure 1:**
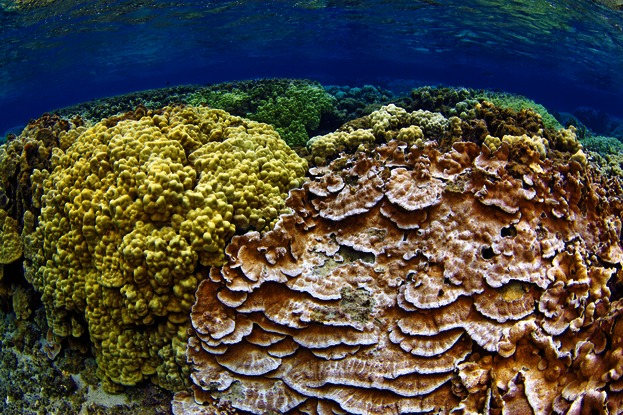
Photo of the coral species *Montipora capitata* (right) and *Porites lobata* (left) from Hawaii. Photo courtesy of Keoki Stender.

**Figure 2:**
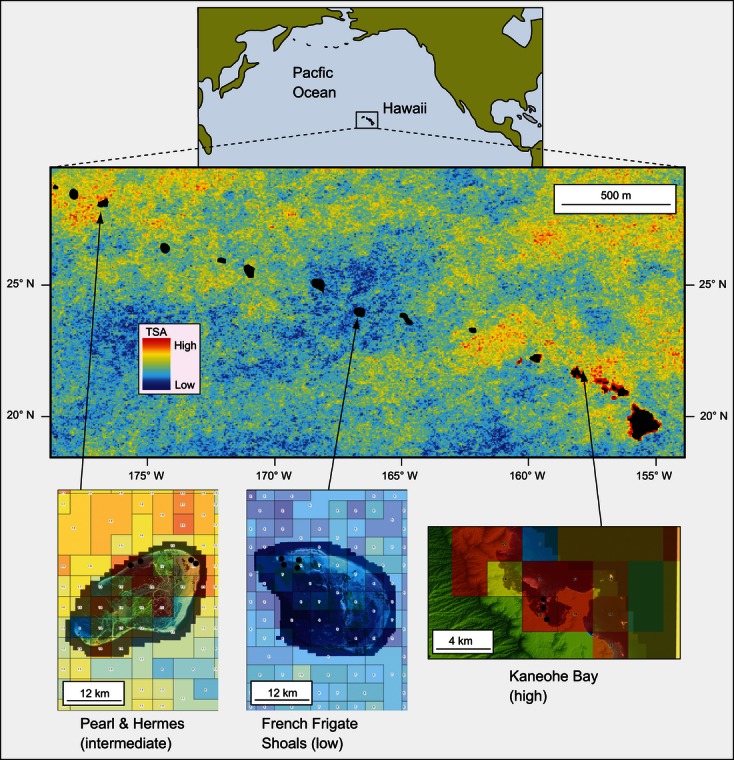
Location of study sites in the Hawaiian archipelago where corals were collected for *Symbiodinium* genotyping. The frequencies of cumulative TSAs between 1985 and 2005 are relatively low at French Frigate Shoals, intermediate at Pearl and Hermes, and high in Kaneohe Bay. Black dots represent sampling sites.

Colonies of *M. capitata* (*n* = 126), *P. lobata* (*n* = 77), and *P. compressa* (*n* = 39) were sampled from Hawaii for *Symbiodinium* genotyping in 2007 (Fig. 1, Table S1). Coral biopsies (≍5 mm²) were collected from each coral from three sites at Kaneohe Bay during June and from four sites each at French Frigate Shoals and Pearl and Hermes during August (Fig. 2). These locations were chosen because they represent a range of cumulative TSAs over the 21-year period between 1985 and 2005, with French Frigate Shoals being the lowest (TSA = 7 and 9), Pearl and Hermes intermediate (TSA = 20 and 21), and Kaneohe Bay the highest (TSA = 36; Selig et al. 2010). Temperature values used in this study were from a 21-year dataset of weekly temperature anomalies using the National Oceanic and Atmospheric Administrations' (NOAA) National Oceanographic Data Center (NODC) Coral Reef Temperature Anomaly Database (CoRTAD) version 1.0 (available at http://www.nodc.noaa.gov/satellitedata/cortad). These data are the longest record of sea surface temperature at the highest resolution globally. The CoRTAD was created from the NODC and University of Miami's Rosentiel School of Marine and Atmospheric Science Pathfinder version 5.0 temperature data (Casey et al. 2010; Selig et al. 2010). Details on methodology for creating the CoRTAD can be found in Selig et al. (2010). All corals were sampled between 1 and 8 m depths from lagoonal habitats, and the coral biopsies stored at 4°C in 400 mL of DNA extraction buffer (50% [w/v] guanidinium isothiocyanate; 50 mmol/L Tris pH 7.6; 10 mmol/L EDTA; 4.2% [w/v] sarkosyl; 2.1% [v/v] β-mercaptoethanol) at the time of collection until further processing.

### DNA extraction, PCR amplification, cloning, and sequencing

Coral biopsies in DNA extraction buffer were incubated at 72°C for 10 min and centrifuged at 16,000*g* for 5 min. The supernatant was mixed with an equal volume of 100% isopropanol to precipitate the DNA and chilled at −20°C overnight. The precipitated DNA was pelleted by centrifugation at 16,000*g* for 15 min, and washed in 70% ethanol before resuspension and stored in Tris buffer (0.1 mol/L pH 8). The *Symbiodinium* partial 5.8S, ITS2, and partial 28S region was amplified in PCR using the forward its-dino (5′-GTGAATTGCAGAACTCCGTG-3′) and reverse its2rev2 (5′-CCTCCGCTTACTTATATGCTT-3′) primers (Pochon and Gates 2010). The products of these amplifications are referred to from here as *Symbiodinium* ITS2 sequences. Each 25-μL PCR reaction contained 1 μL of DNA template, 2.5 μL of 10× ImmoBuffer (Bioline, MA), 0.1 μL IMMOLASE™ Hot-Start DNA Polymerase (Bioline, MA), 3 mmol/L of MgCl_2_, 0.5 μL of 10 mmol/L total dNTPs (2.5 mmol/L each), 5 pmol each primer, and deionized sterile water to volume. PCR was performed on a BioRad (Hercules, CA) iCycler™using the following conditions: 95°C for 7 min, followed by 35 cycles of 45 sec at 95°C, 45 sec at 52°C, and 45 sec at 72°C, with a final extension at 72°C for 5 min. PCR amplicons were purified using the QIAquick® PCR Purification Kit (Qiagen, CA), ligated into the pGEM®-T Easy vector (Promega, WI), transformed into α-select gold efficiency competent cells (Bioline, MA), and grown overnight on selective LB media (ampicillin 50 mg/mL, 0.1 mmol/L IPTG, 50 mg/mL X-gal). Colonies containing the target insert were amplified using M13 primers as in Stat et al. (2009b). PCR products from clones were sequenced using BigDye Terminators (PerkinElmer, MA) on an ABI-3100 automated sequencer at the University of Hawaii.

### Sequence analysis, operational taxonomic units, and statistical parsimony

*Symbiodinium* ITS2 sequences from this study were inspected, edited using MacVector® 11.1, and aligned to the GeoSymbio (Franklin et al. 2012) ITS2 clade alignments. Sequences D1.1 and D1.2 isolated from the soritid foraminifera *Marginopora vertebralis* and the sponge *Haliclona koremella* (see Pochon et al. 2007), respectively, were removed from the clade D alignment, as they are so different from the other clade D sequences that they impede the alignment. Mothur (Schloss et al. 2009) was used to identify unique sequences, generate a distance matrix with each gap treated as a mutation, and group sequences into operational taxonomic units (OTUs) based on 97% sequence similarity using the furthest neighbor algorithm in Hcluster for each *Symbiodinium* clade. OTU clustering generally utilizes sequence similarity values ranging from 95% to 99% (Horner-Devine et al. 2004). We chose a threshold of 97%, as it is the most widely used similarity cutoff (e.g., Bjorbækmo et al. 2010; Brazelton et al. 2010).

Statistical parsimony networks of *Symbiodinium* ITS2 sequences were inferred using the software TCS 1.21 with gaps treated as a fifth state and the connection limit relaxed so that a single network for each clade could be constructed (Clement et al. 2000). For each OTU, a representative sequence (the ancestral sequence) was recovered by performing individual networks. The representative sequences were then used to construct clade networks. Novel sequences identified as representative of an OTU were named by using the closest ITS2 type followed by a decimal and integer (e.g., C15.x) as in Stat et al. (2009b, 2011).

### Statistical analyses

The proportion of coral colonies with and without *Symbiodinium* clade D was compared at three TSA levels (high, intermediate, and low) with two-tailed Fisher's exact tests. Tests were performed independently for each coral host genera. Fisher's exact tests were performed in the software package R (R Development Core Team 2009). The square root of the relative frequency of *Symbiodinium* OTUs present in each colony was also compared using the Bray–Curtis coefficient of similarity (*S*) in the software package PRIMER v.6 (Clarke and Gorley 2006). To test for the partitioning of OTUs by TSA, a permutational multivariate analysis of variance (MANOVA) (Anderson 2001, 2005; McArdle and Anderson 2001) was performed with a design of sites nested within TSA levels and genus as an additional factor. The test was performed using type 3 sums of squares and unrestricted permutation of raw data. The Bray–Curtis similarity of sites was visualized using two-dimensional nonmetric multidimensional scaling (nMDS) and UPGMA clustering, and the SIMPER test was used to determine *Symbiodinium* OTUs that contributed toward the dissimilarity among groups (Clarke 1993).

## Results

### *Symbiodinium* diversity

**Figure 3:**
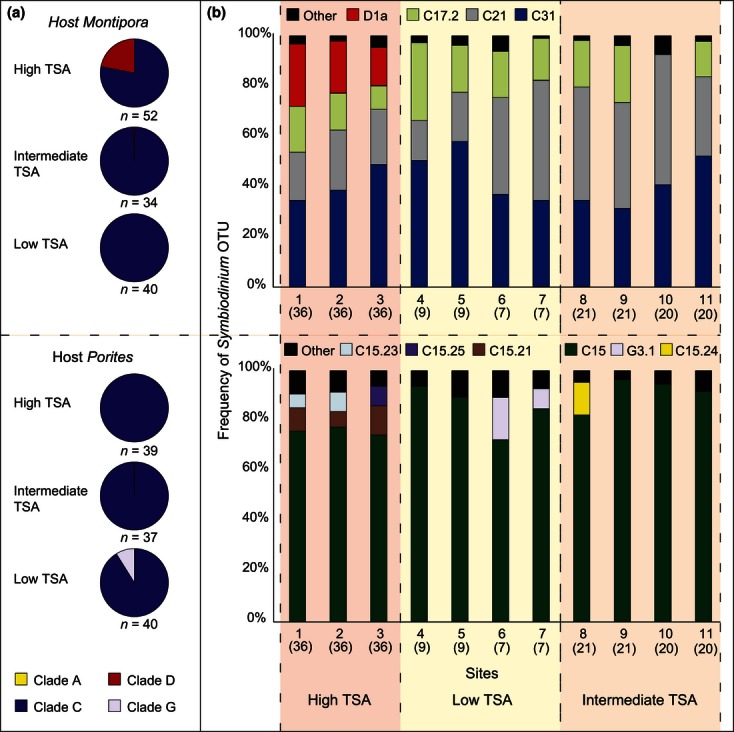
Frequencies of *Symbiodinium* (a) clades and (b) OTUs from colonies of *Montipora* and *Porites*. Pie charts represent *Symbiodinium* clade frequency per region from *n* number of colonies sampled. Histograms represent the frequency of *Symbiodinium* ITS2 OTUs at the 97% similarity cutoff for each site sampled in the study. “Other” represents the cumulative frequency of low abundant (<5%) OTUs. Site numbers within each TSA region are indicated on the *x*-axis with the TSA value in brackets. OTU, operational taxonomic unit; TSA, thermal stress anomalies.

**Figure 4:**
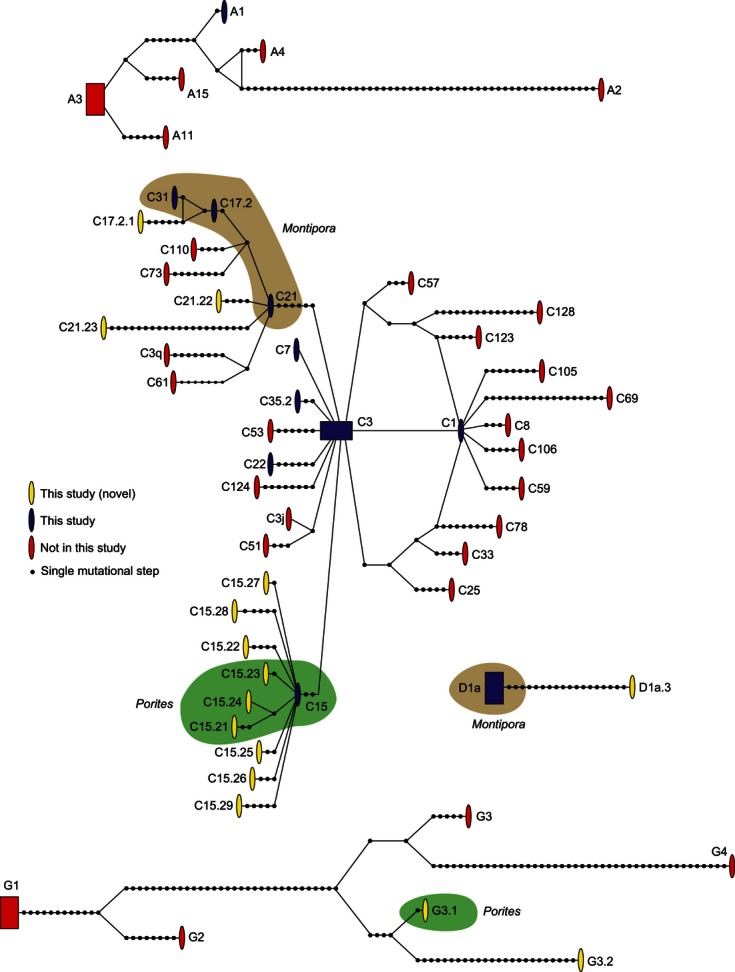
Statistical parsimony networks of *Symbiodinium* operational taxonomic units (OTUs). Each network represents a different clade (clades A, C, D, and G). Rectangles represent the inferred ancestral sequence in the network. Green and brown bubble plots identify abundant OTUs (>5%) associated with *Porites* and *Montipora*, respectively.

A total of 2305 *Symbiodinium* ITS2 sequences were recovered from colonies of *M. capitata*, *P. compressa*, and *P. lobata*. *Symbiodinium* sequences belonging to clades C (*n* = 1010 sequences, 94.7% from 117 colonies) and D (*n* = 57, 5.3% from 13 colonies) were identified from *Montipora* (*n* = 1067 sequences from 126 colonies), and clades A (*n* = 1, 0.1% from one colony), C (*n* = 1195, 96.5% from 115 colonies), D (*n* = 2, 0.2% from two colonies), and G (*n* = 40, 3.2% from seven colonies) from *Porites* (*n* = 1238 sequences from 116 colonies). Of the 2305 sequences, 683 were unique (clade A = 1, C = 647, D = 18, G = 17) that grouped into 26 OTUs (clade A = 1, C = 21, D = 2, G = 2), of which 12 were associated with *Montipora* and 21 with *Porites* (Figs. 3 and 4; Table S1). Accession numbers for novel sequences representing OTUs were deposited in Genbank (KC597683–KC597697; Table S2). In *Montipora*, *Symbiodinium* ITS2 OTU C31 and C21 were the most common representing 42.0% and 32.2%, respectively, while in *Porites*, C15 was the most common representing 84.2%.

To investigate the evolutionary relationship of the OTUs identified in this study within the context of the global diversity of *Symbiodinium*, the unique sequences were combined with the GeoSymbio ITS2 sequences for clades A, C, D, and G. For clade A, 24 unique sequences grouped into 6 OTUs, 878 clade C sequences into 41 OTUs, 29 clade D sequences into two OTUs, and 20 clade G sequences into six OTUs. The statistical parsimony networks for each of these clades are presented in Figure 4. *Symbiodinium* OTU A3, C3, D1a, and G1 were inferred as ancestral in statistical parsimony networks for clades A, C, D, and G, respectively (Fig. 4). In clade C, the nine novel OTUs derived from C15 were associated with *Porites* only, while the novel OTUs associated with *Montipora* clustered with C21 and C17.2. In clade G, the two novel OTUs that associated with *Porites* formed a monophyletic grouping that was most closely related to G3.

### Partitioning of *Symbiodinium* clade D by TSA

The proportion of *Porites* colonies with *Symbiodinium* clade D did not differ from those colonies without clade D at all TSA levels. The proportion of *Montipora* colonies with *Symbiodinium* clade D was significantly higher than those without clade D at the highest TSA level (Fisher's exact test, *P*-value <0.001).

**Figure 5:**
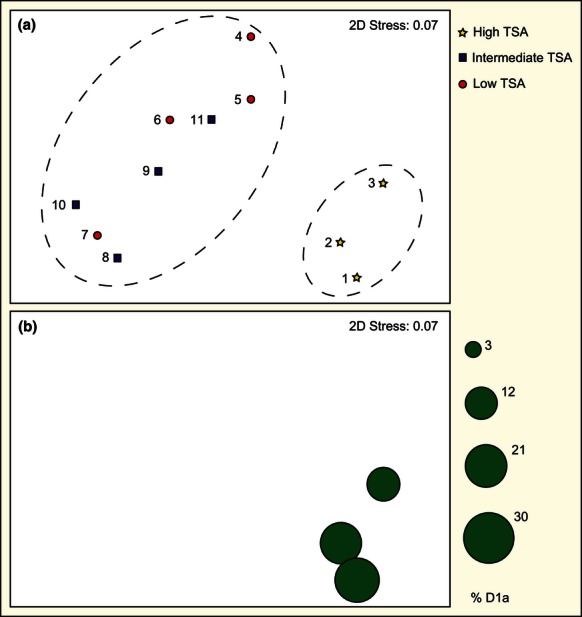
Two-dimensional nonmetric multidimensional scaling (nMDS) plots of *Symbiodinium* operational taxonomic units (OTUs) recovered from *Montipora capitata* grouped by sites 1–11 (a), and bubble plots showing the distribution of *Symbiodinium* OTU D1a (b). The dashed circles in 4a represent the UPGMA cluster groups at the 80% similarity threshold.

To investigate the partitioning of *Symbiodinium* OTUs, a permutational MANOVA was performed (Table 1). *Symbiodinium* OTUs were significantly different between the two coral genera, by TSA regions, and by coral genera × TSA region. As clade D in *Montipora* at the highest TSA level was significant in the Fisher's exact test, and TSA and coral genera × TSA were significant in the permutational MANOVA (Table 1), the correlation between *Symbiodinium* clade D in *M. capitata* and the highest TSA region was explored further. A significant difference was found between the highest TSA region compared with the lowest (*P* = 0.001) and intermediate (*P* = 0.008) regions, but not between the intermediate and lowest TSA regions in pairwise comparisons of *Symbiodinium* OTUs for host *Montipora*. Two-dimensional nMDS with UPGMA clustering at 80% overlaid shows distinct groups for sites located in the highest TSA region (sites 1–3) compared with all other sites (Fig. 5a). Analysis from the SIMPER test identified *Symbiodinium* OTU D1a in the highest TSA region as contributing the most toward the dissimilarity to the intermediate (44.04%) and low (44.77%) TSA regions, and is clearly represented in a bubble plot of the distribution of the D1a OTU (Fig. 5b). Taken together, there is a clear correlation between the highest TSA region and the occurrence of *Symbiodinium* clade D in *Montipora*.

**Table 1:** Permutational MANOVA of *Symbiodinium* OTUs

Source	df	Pseudo-F	*P*
Host genus	1	65.89	0.004*
TSA	2	8.11	0.001*
Site (TSA)	8	1.02	0.518
Host genus × TSA	2	6.44	0.001*
Host genus × Site (TSA)	8	1.14	0.283

MANOVA, multivariate analysis of variance; OTU, operational taxonomic unit; TSA, thermal stress anomalies.

* Significant values (*P* < 0.05).

## Discussion

### Association of *Symbiodinium* clade D with *Montipora* and *Porites*

The algal endosymbiont, *Symbiodinium* clade D associates with corals in the genus *Montipora* but not *Porites* in Hawaii. The occurrence of clade D in *Montipora* correlates with an area that has experienced the highest recorded frequency of TSAs for lagoonal habitats in Hawaii. This spatial distribution suggests that thermal stress may influence the distribution of clade D in this coral. While the cause behind the correlation between clade D and ocean temperature stress remains unclear, a shift in response to recent environmental conditions, notably the frequency of TSAs in the region, is one plausible explanation. A community shift of coral endosymbionts toward a population dominated by clade D in response to elevated ocean temperature stress is consistent with reports from other corals in the Pacific, including *Acropora* and *Pocillopora*, and is consistent with regional reports of an increase in the abundance of clade D on reefs that have recently experienced thermal stress (Baker et al. 2004; Jones et al. 2008; LaJeunesse et al. 2008). Clade D can also be common in corals located in an environment characterized by relatively high ocean temperatures compared with most other regions where corals are found, such as the Persian Gulf where the temperature reaches 33°C (Mostafavi et al. 2007; LaJeunesse et al. 2010; Stat and Gates 2011). As the ocean temperature of Hawaiian reefs do not fall into this category and remain under 30°C (NOAA National Weather Service), and the occurrence of clade D in *Montipora* is extremely rare globally (Franklin et al. 2012), it is unlikely that this association is the result of long-term local adaptation to challenging ocean temperatures, but rather a response to recent TSAs. Furthermore, the annual incidence of TSAs in the years leading up to sampling implies that chronic temperature stress in the absence of bleaching can provide a competitive advantage for clade D *Symbiodinium* to persist in these corals in Kaneohe Bay. This is consistent with the observation on the abundance of clade D increasing in corals during ocean warming prior to bleaching (LaJeunesse et al. 2008). It is also possible that anthropogenic impacts, like pollution and sedimentation, have contributed to the occurrence of clade D *Symbiodinium* in *Montipora* at Kaneohe Bay (Cooper et al. 2011; Stat and Gates 2011). Oahu is the most populated island in Hawaii, and Kaneohe Bay specifically has been exposed to high levels of pollution (Hunter et al. 1995). In contrast, French Frigate Shoals and Pearl and Hermes are located within the Paphānaumokuākea Marine National Monument, a protected marine environment that is arguably one of the least impacted coral reef ecosystems in the world. Future work will focus on the occurrence of clade D on the island of Oahu, and investigate whether TSA or other anthropogenic impacts like pollution, or their synergism, accounts for the higher abundance of clade D in Kaneohe Bay.

Even though clade D sequences were found associated with *Porites* (*n* = 2), the extremely low abundance (0.2% of sequences) could suggest that they represent surface contaminants, although it is impossible to rule out the possibility that they are low abundant endosymbionts (Mieog et al. 2007; Silverstein et al. 2012). In the Pacific, clade D is rare in *Porites* and has only been identified in two colonies from Palau (Fabricius et al. 2004), even though genotyping of its *Symbiodinium* community extends to numerous regions including the southern, central, and northern Great Barrier reef, Johnston Atoll, Japan, Guam, Hawaii, and American Samoa (LaJeunesse et al. 2003, 2004a,b; Apprill and Gates 2007; Stat et al. 2008b; Barshis et al. 2010; Pochon et al. 2010; Franklin et al. 2012). While clade D in *Porites* from Palau originated from colonies in a chronically warm environment, most *Porites* in the study from that location (and others) harbored clade C. Why some corals show flexibility in their symbioses and a shift toward clade D under certain environmental conditions and others do not (Thornhill et al. 2006; LaJeunesse et al. 2007, 2008; Costa et al. 2008; Jones et al. 2008; Stat et al. 2009a; McGinley et al. 2012) remains unclear. The corals used in this study are highly abundant in Hawaii and both occupy similar environments and acquire their symbionts via maternal transmission; however, they show very different affinities for clade D. One explanation may lie in the dependency of the host for their endosymbiotic community. *Porites* and *Montipora* show differences in the dependency for their endosymbiont population, especially during periods of thermal stress (Grottoli et al. 2006; Rodrigues and Grottoli 2007). *Montipora capitata* shifts from autotrophy to heterotrophy during episodes of thermal stress and bleaching. In contrast, *P. compressa* and *P. lobata* are highly autotrophic, do not make a significant transition to heterotrophy, and thus rely on their endosymbiotic population for nutrients during recovery. The switch to heterotrophy in *Montipora* supports a less specific association in *Montipora* and a lower dependency on their endosymbionts, which may partly explain the observed community shift from their dominant C31 symbiont to clade D in areas of high thermal history in Hawaii. However, if the switch to clade D *Symbiodinium* allows corals to adapt to environmental change and increases their thermal tolerance (i.e., symbiont dependence; Jones and Berkelmans 2010) then the concurrent switch to heterotrophy (i.e., symbiont independence) during such conditions is somewhat of a paradox. One explanation is that clade D may provide the host with a reduced amount of nutrients, but enough to supplement the amount acquired through host heterotrophy under periods of stress and collectively equating to the amount needed to sustain the host. This is consistent with the opportunistic nature of clade D and reports of less carbon that is translocated to the host by clade D compared with clade C *Symbiodinium* (Cantin et al. 2009). The corals' tolerance or susceptibility to changes in the environment is therefore a culmination of numerous factors that includes but is not limited to (a) the dynamics of host–symbiont assemblages; (b) the differential survival of symbionts under varying conditions; (c) the contributions of various symbionts to the host; and (d) the dependence of the host for their *Symbiodinium* community and the ability to make a transition to heterotrophy.

### *Symbiodinium* diversity inferred using OTUs

We applied a sequence similarity threshold to group *Symbiodinium* sequences into OTU's to assess diversity. As in other taxa, some OTU groups may represent a species cluster or functional group, while others may combine species or represent subspecies. This inconsistency reflects the biological diversity of organisms and the lack of a uniform genetic divergence that delimits species boundaries. Also, grouping sequences into OTUs based on sequence similarity does not overcome all the problems associated with PCR artifacts and intragenomic variation (Thornhill et al. 2007; Stat et al. 2011). However, these caveats are not limited to *Symbiodinium* and are common across taxa, and this method is a widely utilized approach for analyzing cloned amplicons from environmental populations of prokaryotes, basal eukaryotes (e.g., Landeweert et al. 2003; Bjorbækmo et al. 2010; Brazelton et al. 2010), and more recently to diversity studies using next-generation sequencing in eukaryotes (e.g., Blaalid et al. 2012).

Statistical parsimony networks were constructed using representative sequences from each OTU for the *Symbiodinium* clades that were identified in this study (A, C, D, and G). As expected, the relationship among OTUs is similar to the phylogenies constructed using ITS2 types identified using the dominant band in DGGE fingerprints (Pochon et al. 2007; LaJeunesse et al. 2008), but with a reduction in complexity. Furthermore, a comparison of the OTUs and their evolutionary relationship compared with the “species clusters” in ITS2 networks identified by Correa and Baker (2009) using a different method are very similar. This study utilizes more sequence data to infer OTUs as an outcome of increased diversity discovered since the Correa and Baker analysis in 2009 and the incorporation of cloning data that increases the likelihood of detecting low abundant symbionts and/or intragenomic variation. This added sequence data likely contributes to the differences in the number of OTU's identified in this study (A:6, C:41, D:2) and the number of “species clusters” that were inferred by Correa and Baker (2009; A:7, C:23, D:1). As the diversity of *Symbiodinium* observed increases, coupled with the amount of genetic data that will likely flood future analysis due to next-generation sequencing platforms, cluster-based approaches to infer *Symbiodinium* diversity will be a necessity.

### *Symbiodinium* diversity in *Montipora* and *Porites*

As with the majority of corals in the Pacific, *Porites* and *Montipora* predominantly associate with clade C *Symbiodinium*, while clade D is occasionally found in *Montipora* (LaJeunesse 2005; Stat et al. 2009b, 2011; Franklin et al. 2012; this study). Furthermore, *Porites* and *Montipora* show specificity with *Symbiodinium* strains within clade C. In *Porites*, endosymbionts belonging to the C15 or the C15-like symbiont cluster are found throughout the Pacific (LaJeunesse et al. 2003, 2004a,b; LaJeunesse 2005; Stat et al. 2008b, 2009b; Barshis et al. 2010; but see Wicks et al. 2010). This ubiquitous distribution of a specific host–symbiont association over a large biogeographic area infers a long-standing association that has developed over evolutionary timescales. Interestingly, *Montipora* predominantly associates with C31 in Hawaii, but in the Great Barrier Reef it associates with C31, and C15 – the symbiont found in *Porites* throughout the Pacific (LaJeunesse et al. 2004a; Stat et al. 2008b). Why C15 associates with *Porites* but not *Montipora* in Hawaii remains unknown. In addition, novel *Symbiodinium* OTUs in *Porites* that were not found in *Montipora* (C15.21–C15.29, Fig. 3) form a monophyletic group with C15 at the root. This implies that an intimate association between C15 and *Porites* in the remote Hawaiian Islands is providing the opportunity for the radiation of new symbiont lineages in the C15 cluster that is specific to this host.

A paradox that exists in the specificity of coral–algal symbioses becomes evident when extending the observed interactions beyond clade C. Coral hosts belonging to a variety of genera show specificity to unique *Symbiodinium* types or lineages within clade C (e.g., *Montipora* and C31, *Pocillopora* and C42; LaJeunesse et al. 2004a). The same host genera, however, are also found in unions with different *Symbiodinium* clades, specifically clades A and D (LaJeunesse et al. 2007; Stat et al. 2009b). Therefore, while there is apparent specificity among closely related symbionts within clade C, the barrier to specificity breaks down among clades. The *Symbiodinium* associations found in this study also support these observations. In addition to the specificity between clade C *Symbiodinium* and the hosts *Montipora* and *Porites*, clade D was found associated with *Montipora*, while clades A, D, and G were found associated with *Porites*. While clade A (and clade B) can be the dominant symbiont in *Porites* from the Caribbean (Thornhill et al. 2006; Finney et al. 2010), its occurrence in *Porites* in the Pacific is extremely rare. As only a single clade A sequence was recovered, the occurrence of this *Symbiodinium* lineage in *Porites* likely represents a surface contaminant or low abundant endosymbiont, like clade D in *Porites*. The association between *Porites* and clade G *Symbiodinium* at French Frigate Shoals is a very interesting observation. This lineage of *Symbiodinium* is usually found in Foraminifera, sponges and soft corals (van Oppen et al. 2005; Pochon et al. 2007; Granados et al. 2008; Hill et al. 2011), and has only been found to associate with single colonies of the corals *Coeloseris* and *Montastraea* in the Indian Ocean (LaJeunesse et al. 2010). Interestingly, *Porites* are often bioeroded by sponges that associate with clade G *Symbiodinium*, and the shared interaction with clade G by these hosts may reflect this three-way interaction (Sammarco and Risk 1990; Granados et al. 2008).

This study presents evidence for the differential association of the algal endosymbiont *Symbiodinium* clade D and two dominant corals in Hawaii. While clade D can occur as the dominant symbiont in *Montipora*, it is nearly absent in *Porites*. Furthermore, the distribution of clade D correlates with the region that has experienced the greatest history of thermal stress, providing additional evidence for the observation of this *Symbiodinium* lineage in areas where ocean conditions are challenging for corals or have recently experienced ocean warming. This study also adds to the accumulating evidence for the interaction of multiple *Symbiodinium* clades with hosts that have been perceived as forming specific symbioses, and calls for a reassessment of what defines specificity in coral–algal symbioses (Silverstein et al. 2012). The difference in abundance and distribution of clade D, and the presence of multiple clade lineages in addition to the dominant-specific symbiont of these corals (i.e., C15 for *Porites* in Hawaii, and C31 for *Montipora*) highlight the biological complexity of these unions. The dependence of the host for their endosymbiotic community, the contribution of different *Symbiodinium* clades and subclades to their host, and how changes in the environment effect these interactions will be a focus of future research investigating the adaptive potential of corals.

## Supporting Information

**Table S1.** Coral colonies, location, and their associated Symbiodinium OTUs.

**Table S2.** *Symbiodinium* sequences and accession numbers.
